# Identification of genetic interactions with *priB* links the PriA/PriB DNA replication restart pathway to double-strand DNA break repair in *Escherichia coli*

**DOI:** 10.1093/g3journal/jkac295

**Published:** 2022-11-03

**Authors:** Aidan M McKenzie, Camille Henry, Kevin S Myers, Michael M Place, James L Keck

**Affiliations:** Department of Biomolecular Chemistry, University of Wisconsin School of Medicine and Public Health, Madison, WI 53706, USA; Department of Biochemistry, University of Wisconsin-Madison, Madison, WI 53706, USA; Great Lakes Bioenergy Research Center, University of Wisconsin-Madison, Madison, WI 53726, USA; Great Lakes Bioenergy Research Center, University of Wisconsin-Madison, Madison, WI 53726, USA; Department of Biomolecular Chemistry, University of Wisconsin School of Medicine and Public Health, Madison, WI 53706, USA

**Keywords:** DNA replication restart, *Tn*-seq, *priA*, *priB*, *priC*, *dnaT*, double-strand DNA breaks, MuGam-GFP

## Abstract

Collisions between DNA replication complexes (replisomes) and impediments such as damaged DNA or proteins tightly bound to the chromosome lead to premature dissociation of replisomes at least once per cell cycle in *Escherichia coli*. Left unrepaired, these events produce incompletely replicated chromosomes that cannot be properly partitioned into daughter cells. DNA replication restart, the process that reloads replisomes at prematurely terminated sites, is therefore essential in *E. coli* and other bacteria. Three replication restart pathways have been identified in *E. coli*: PriA/PriB, PriA/PriC, and PriC/Rep. A limited number of genetic interactions between replication restart and other genome maintenance pathways have been defined, but a systematic study placing replication restart reactions in a broader cellular context has not been performed. We have utilized transposon-insertion sequencing to identify new genetic interactions between DNA replication restart pathways and other cellular systems. Known genetic interactors with the *priB* replication restart gene (uniquely involved in the PriA/PriB pathway) were confirmed and several novel *priB* interactions were discovered. Targeted genetic and imaging-based experiments with *priB* and its genetic partners revealed significant double-strand DNA break accumulation in strains with mutations in *dam*, *rep*, *rdgC*, *lexA*, or *polA*. Modulating the activity of the RecA recombinase partially suppressed the detrimental effects of *rdgC* or *lexA* mutations in Δ*priB* cells. Taken together, our results highlight roles for several genes in double-strand DNA break homeostasis and define a genetic network that facilitates DNA repair/processing upstream of PriA/PriB-mediated DNA replication restart in *E. coli*.

## Introduction

Cell propagation relies on high-fidelity genome duplication. To accomplish this task, DNA replication complexes (replisomes) loaded onto origins of replication traverse the genome, utilizing parental DNA as templates as they synthesize new DNA strands. During this process, replisomes frequently collide with obstacles such as DNA damage or nucleo-protein complexes. In the most severe instances, these encounters cause replisomes to dissociate from the genome. In *Escherichia coli*, it is estimated that at least once per cell cycle a replisome prematurely dissociates from the chromosome (Cox *et al.* 2000; Mangiameli *et al.* 2017). Bacteria have therefore evolved mechanisms to reload replisomes at premature replication termination sites so that cells can complete genome duplication processes (Michel and Sandler 2017; Windgassen, Wessel, *et al.* 2018).

**Fig. 1.**
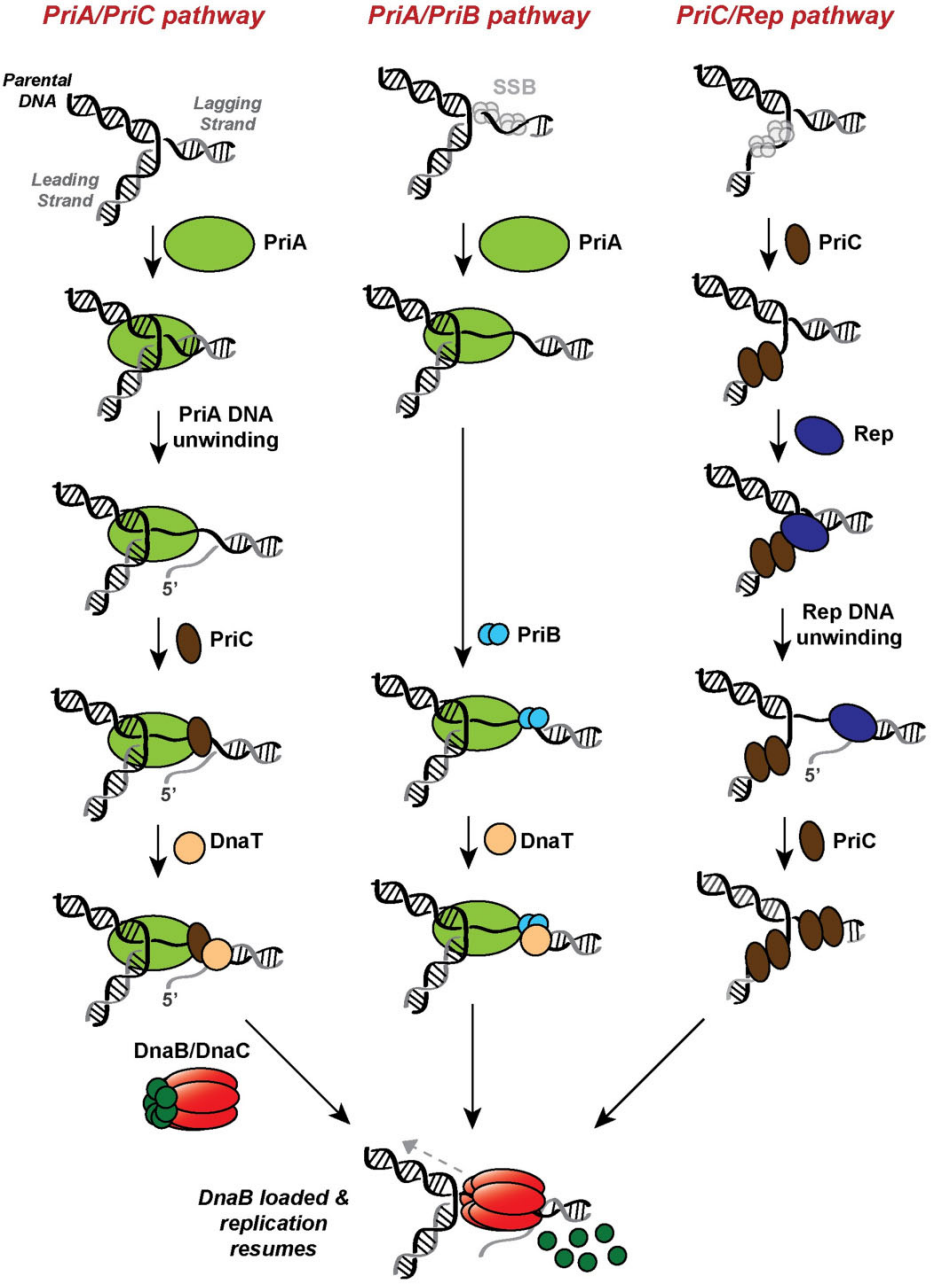
Pathways of DNA replication restart in *E. coli*. PriA/PriC (left) and PriA/PriB (center) pathways efficiently recognize abandoned fork substrates with nascent leading strands, while the PriC/Rep (right) pathway prefers fork substrates with a leading strand gap. All 3 pathways recognize an abandoned fork, remodel the substrate (if needed) and recruit other replication restart proteins, and load the replicative helicase (DnaB) with the help of the helicase loader (DnaC) to restart DNA replication. The PriA/PriB pathway (center) is inactivated in Δ*priB* cells, the PriA/PriC (left) and PriC/Rep (right) pathways are inactivated in *priC::kan* cells, and the PriA/PriC (left) pathway is inactivated in *priA300* mutants.

Genetic and biochemical studies have defined three pathways of DNA replication restart in *E. coli*: PriA/PriB, PriA/PriC, and PriC/Rep (Fig. 1) (Lee and Kornberg 1991; Nurse *et al.* 1991; Masai *et al.* 1994; Sandler 2000; Sandler *et al.* 2001; McCool, Ford, *et al.* 2004; Heller and Marians 2005a; Manhart and McHenry 2013; Sandler *et al.* 2021). Null mutations in *priA* or *dnaT* cause similar severe phenotypes, and both genes have been placed in the PriA/PriB and PriA/PriC pathways (Lee and Kornberg 1991; Nurse *et al.* 1991; Masai *et al.* 1994; McCool, Ford, *et al.* 2004). Conversely, minor phenotypes associated with mutations in *priC* or *rep* have placed them in the less frequently utilized PriC/Rep pathway, independent of PriA. *priB* or *priC* can each be deleted independently, but simultaneous deletion of both genes deactivates all three DNA replication restart pathways, resulting in lethality. In addition, a mutation encoding an ATPase- and helicase-deficient variant of PriA (*priA300*) elicits severe defects when paired with a *priB* deletion, but not a *priC* deletion (Sandler *et al.* 2001). Therefore, PriA helicase activity is likely required to facilitate the PriA/PriC pathway, but not the PriA/PriB pathway (Fig. 1). Each restart pathway recognizes abandoned DNA replication forks, remodels the forks to allow replisome loading, and reloads the replicative helicase (DnaB) with the help of its helicase loader (DnaC). After DnaB is reloaded, it recruits the remaining members of the replisome via protein–protein interactions (Tougu *et al.* 1994; Kim *et al.* 1996a,b; Costa *et al.* 2013).

Evidence suggests that different replication restart pathways can be preferentially utilized and/or that each operates on distinct substrates. For example, the PriA/PriB restart pathway appears to be favored following DNA recombination (Sandler *et al.* 1999). Mutations in *priB* are also more detrimental than *priC* when paired with a *holD* mutation, which increases instances of fork stalling and collapse (Flores *et al.* 2002). These results could indicate a heavier reliance on PriA/PriB than other pathways for replication restart. In addition, a *priB* deletion is synthetically lethal with mutations in *dam*, which encodes a DNA methyl transferase whose absence is linked to increased double-strand DNA breaks (DSBs) (Marinus 2000; Nowosielska and Marinus 2005; Boonsombat *et al.* 2006). This observation suggests that PriA/PriB replication restart could be important following DSB repair. Although *priC* disruption alone results in negligible phenotypic effects, in vitro evidence suggests that abandoned replication forks with long single-stranded (ss) DNA gaps between the nascent leading strand and parental duplex DNA may be recognized and remodeled efficiently by the PriC/Rep pathway, which could indicate its preference for specific abandoned DNA replication fork structures (Fig. 1) (Heller and Marians 2005a).

Candidate-based genetic studies have uncovered a limited number of genes linked to DNA replication restart, but a systematic study examining the potential importance of all genes as they relate to this process is lacking. Motivated by the idea that finding novel genetic interactions with each DNA replication restart pathway could help place each in a broader cellular context, we used transposon-insertion sequencing (*Tn*-seq; Langridge *et al.* 2009; van Opijnen *et al.* 2009; van Opijnen and Camilli 2013; Barquist *et al.* 2016) in Δ*priB*, *priC::kan*, and *priA300 E. coli* strains to identify genes that are conditionally important in each strain. Deletion of *priB* inactivates the PriA/PriB pathway, deletion of *priC* inactivates the PriA/PriC and PriC/Rep pathways, and the *priA300* allele disables the PriA/PriC pathway (Fig. 1) (Sandler 2000; Sandler *et al.* 2001; Windgassen, Wessel, *et al.* 2018; Sandler *et al.* 2021). The Δ*priB Tn*-seq screen yielded particularly informative results whereas the *priC::kan*, and *priA300* screens yielded far fewer hits, consistent with the PriA/PriB pathway serving as the primary replication restart mechanism in *E. coli*. The screen and additional genetic experiments corroborated prior genetic results in which *priC*, *rep*, and *dam* are conditionally essential or important in Δ*priB* cells. Strikingly, the screen also identified many new interactions between *priB* and genes involved in genome maintenance (*lexA*, *rdgC*, *uup*, *rdgB*, and *polA*) and other processes (*nagC*). Mutations in many of these genes produced strong growth defects in Δ*priB* cells, evidenced by plasmid retention, growth competition, and spot plating assays. Furthermore, *rep*, *lexA*, *polA*, and *dam* mutants were hypersensitive to ciprofloxacin, which induces DSBs. These mutant strains also accumulated DSBs in vivo and displayed significant cell filamentation, a common indicator of poor genomic maintenance. Lastly, some of the toxicity to Δ*priB* cells caused by mutations in *lexA* or *rdgC* appears to result from inappropriate and/or excessive RecA recombinase activity. These results highlight the importance of several genes in Δ*priB E. coli*, strengthen experimental evidence of the connection between the PriA/PriB restart pathway and DSB repair, and help elucidate the interplay between DNA repair and DNA replication restart processes.

## Materials and methods

### Strain construction

All strains used in this study are derivatives of *E. coli* MG1655 (Supplementary Table 1). To enhance the viability and ease of cloning, all strains (unless otherwise stated in Supplementary Table 1) carry the *sulB103* allele, encoding an FtsZ variant that resists SulA-mediated cell division inhibition (Bi and Lutkenhaus 1990; McCool, Long, *et al.* 2004). All plasmids and oligonucleotides used in this study are listed in Supplementary Table 2. To construct derivative *polA12(ts)* and MuGam-GFP strains, the method developed by Datsenko and Wanner (2000) was employed with some modifications, as described previously (Romero, Chen, *et al.* 2020). All strains constructed with P1 transduction utilized kanamycin selection, many of which relied on Keio collection strains as donors (Baba *et al.* 2006). Sources of strains and plasmids are provided in Supplementary Tables 1 and 2 (Singer *et al.* 1989; Cherepanov and Wackernagel 1995; Huang *et al.* 1997; Sandler *et al.* 1999; Datsenko and Wanner 2000; Bernhardt and de Boer 2004; Boonsombat *et al.* 2006; Shee *et al.* 2013; Byrne *et al.* 2014; Kim *et al.* 2015; Henrikus *et al.* 2019; Romero, Chen, *et al.* 2020). All chromosomal mutations were confirmed with PCR amplification flanking the locus of interest and, if necessary, verified with Sanger sequencing. We note that attempts to disrupt *priB* in two genome-wide gene replacement studies have suggested that *priB* may be essential in *E. coli* (Baba *et al.* 2006; Goodall *et al.* 2018). However, *priB* has been successfully deleted in *E. coli* when deletion is carried out in a manner that does not perturb expression of genes downstream of *priB* within its operon (Sandler *et al.* 1999; Sandler 2000). One of the two downstream genes encode an essential ribosomal protein gene (*rpsR*). The *priB* deletion allele that has been used successfully in prior experiments (*del(priB)302*) is used here (Supplementary Table 1).

### Transposome preparation

Transposon mutagenesis was performed using the EZ-Tn5 <DHFR-1> transposon kit (Epicentre) and EK54/MA56/LP372 Tn5 transposase, a hyperactive variant (Goryshin and Reznikoff 1998). The Tn5 transposon was PCR amplified with oAM054 and Phusion polymerase (New England Biolabs). Tn5 transposase was purified as described previously (Bhasin *et al.* 1999; Byrne *et al.* 2014). Transposomes were prepared by incubating 2.5 pmol of Tn5 DNA with 0.5 nmol of Tn5 transposase in 20 µl for 3 h at room temperature before dialyzing into 1× TE for 3 h to remove salt prior to electroporation.

### Generation of electrocompetent cells and in vivo transposition

*E. coli* strains were prepared for transposition as previously described (Byrne *et al.* 2014). Briefly, cells in mid-log phase were washed 3 times with ice-cold 10% v/v glycerol. In the final wash, cells were either resuspended in 10% v/v glycerol or glycerol-yeast extract medium (10% v/v glycerol, 0.125% w/v yeast extract, and 0.25% w/v tryptone), flash frozen with liquid nitrogen, and stored at −80°C. Dialyzed transposome (5 µl) was mixed with 100 µl of electrocompetent cells, electroporated, and immediately recovered in 1 ml of SOC medium (2% w/v tryptone, 0.5% w/v yeast extract, 0.05% w/v NaCl, 2.5 mM KCl, 10 mM MgCl_2_, and 20 mM glucose) for 1 h. After recovery, dilutions of the cells were plated on Super Optimal Broth (SOB)-agar (2% w/v tryptone, 0.5% w/v yeast extract, 0.05% w/v NaCl, 2.5 mM KCl, 1.5% w/v agar, 10 mM MgCl_2_, and 20 mM MgSO_4_) containing 10 µg/ml trimethoprim to select for transposon-insertion mutants. Colony counts for each library were estimated by counting one-third of ∼10% of plates. To pool the mutants and construct libraries of ∼500,000 insertion mutants, 2 ml of Luria Broth (LB) (1% w/v tryptone, 0.5% w/v yeast extract, and 1% w/v NaCl) was added to each plate to scrape the colonies into a thick slurry. Care was taken to sufficiently mix each slurry before archiving each in technical triplicate (in 50% glycerol) at −80°C.

### Preparation of transposon-insertion DNA for sequencing

For sufficient sampling, 100 ml of LB (with 10 µg/ml trimethoprim) was inoculated to OD_600_ ∼0.02 with each respective transposon-insertion mutant library and grown overnight at 37°C. Genomic DNA was purified using a Wizard Genomic DNA Purification Kit (Promega) and quantified using the QuantiFluor ONE dsDNA System (Promega). Genomic DNA was sheared to ∼200-bp fragments with sonication. The resulting gDNA fragments were prepared for sequencing using NEBNext Ultra II DNA Library Prep Kit for Illumina (New England Biolabs). Bead-based size selection was used to enrich for 200-bp fragments prior to a 21-cycle splinkerette PCR utilizing a custom Tn5-enriching forward primer (oAM055) and custom indexed reverse primers for multiplexing (oAMrev) (Barquist *et al.* 2016). To ensure the quality and length of amplified DNA, a final bead-based size selection was employed. DNA was then sequenced with a NextSeq platform (Illumina) at the University of Michigan Advanced Genomics Core using a custom read primer (oAM058) to read the last 10 nt of the transposon before entering chromosomal DNA (to ensure reads corresponded to Tn5 insertions). To maintain sufficient sequence diversity on the flow-cell, a phiX174 DNA spike (20%) was also included in the run. A custom index read primer (oAM059) and standard Illumina primer (oAM112) were employed for sequencing the read indexes and PhiX174 DNA, respectively.

### *Tn*-seq data analysis

*Tn*-seq sequencing files were trimmed with fastx_trimmer.pl version 0.0.13.2 (http://hannonlab.cshl.edu/fastx_toolkit) using default parameters except the first base to keep (-f flag) was set to 10 to remove transposon sequence. Individual samples were then split with fastx_barcode_splitter.pl, version 0.0.13.2 (http://hannonlab.cshl.edu/fastx_toolkit) using a file containing the sample ID and the individual barcode sequence used to split each sample into an individual FASTQ file. The barcode sequence was then removed from each read within each FASTQ file using Cutadapt, version 1.13 (Martin 2011). The trimmed FASTQ files were then aligned to the *E. coli* K-12 MG1655 genome (NC_000913.3) using Bowtie2, version 1.2 using default parameters (Langmead and Salzberg 2012). Conditionally important or essential genes were determined using TSAS, version 0.3.0 using Analysis_type2 for 2-sample analysis to compare transposon-insertion profiles of each mutant strain to the *wt* (Burger *et al.* 2017). Weighted read ratios were calculated as described previously (Burger *et al.* 2017). All other parameters were kept at the default settings. *Tn*-seq analysis is included Supplementary File 1.

### Plasmid (*priB*-pRC7) retention assay

The *priB*-pRC7 plasmid is a lac^+^ mini-F (low-copy) derivative of pFZY1 (Bernhardt and de Boer 2004) containing the *priB* gene. PCR amplification of *priB* with oAM170 and oAM171 conferred ApaI restriction sites flanking the gene. The resulting PCR product and the empty pRC7 plasmid were digested with ApaI and ligated, yielding *priB*-pRC7. Gene deletions via P1 transduction were carried out after the cells had been transformed with the *priB*-pRC7 plasmid to help ensure the viability of each mutant tested. Once constructed, cultures were grown overnight in LB supplemented with 50 µg/ml ampicillin. The following day, cells were diluted 100× in LB and grown to ∼0.2 OD_600_ shaking at 37°C. The cultures were then placed at 4°C, serially diluted, and plated on SOB-agar containing X-gal (80 µg/ml) and IPTG (1 mM) to yield 50–500 colonies per plate. Most colonies were counted and imaged after 16 h incubations at 37°C, but plates used in Fig. 7 were incubated for 22 h to better visualize the small white colonies. Colony counts and analysis are included in Supplementary File 2.

### Growth competitions

A growth competition experiment was used to determine if deleting *rdgB* conferred a measurable fitness defect in Δ*priB* cells. Pairwise competitions were constructed where the fitness effect of a Δ*rdgB* mutation was examined in a *priB*^+^ or Δ*priB* strain. To quantify the abundance of the Δ*rdgB* mutant, one strain within each competition was modified to carry a neutral Δ*araBAD* mutation. When *ara^−^* or *ara*^+^ strains are plated on medium containing tetrazolium and arabinose, they form red or white colonies, respectively. The individual strains of each competition were grown in isolation overnight at 37°C in LB, and then, equivalent volumes of each were mixed and diluted 100× in fresh LB. The cultures (now with competing strains) resumed growth at 37°C, and incubations were temporarily paused every 24 h to re-dilute (100×) in fresh LB and quantify the Δ*rdgB* mutant abundance by plating on LB agar (1% w/v tryptone, 0.5% w/v yeast extract, 1% w/v NaCl, and 0.75% w/v agar) with tetrazolium (0.005% w/v) and arabinose (1% w/v). The competitions were performed in biological triplicate and with pairwise alternation of the Δ*araBAD* mutation (to ensure it did not produce a fitness effect). Colony counts and analysis are included in Supplementary File 2.

### Spot plating experiments

Serial dilution spot plating was used to examine mutant sensitivities to ciprofloxacin and the effect of temperature and media on *polA12(ts)* strains. For ciprofloxacin sensitivity experiments, biological triplicate LB cultures were inoculated and grown overnight at 37°C, whereas strains used in the *polA12(ts)* experiment were grown at 30°C. The following day, the cultures were diluted to OD_600_ of 1.0 and 10× serial dilutions were prepared with LB or M9 (0.6% w/v Na_2_HPO_4_, 0.3% w/v KH_2_PO_4_, 0.05% w/v NaCl, 0.1% w/v NH_4_Cl, 1 mM MgSO_4_, 0.1 mM CaCl_2_, and 0.1% w/v glucose) media. Serial dilutions (10 µl) ranging from 10^−1^ to 10^−6^ were spot plated and incubated at 37°C, unless stated otherwise. LB agar plates were incubated for 16 h, and M9 agar (0.6% w/v Na_2_HPO_4_, 0.3% w/v KH_2_PO_4_, 0.05% w/v NaCl, 0.1% w/v NH_4_Cl, 1 mM MgSO_4_, 0.1 mM CaCl_2_, 0.1% w/v glucose, and 1.6% w/v agar) plates were incubated for 40 h before imaging.

### Fluorescence and brightfield microscopy

An *E. coli* strain carrying MuGam-GFP (SMR14334; Shee *et al.* 2013) was derivatized to carry the *sulB103* allele (*wt*) before P1 transduction deleted other genes of interest. Saturated cultures were diluted 100× and grown in LB for 30 min at 37°C to enter early exponential phase. MuGam-GFP expression was then induced at 100 ng/ml doxycycline and growth continued for an additional 2.5 h at 37°C. Cells were pelleted and resuspended in 1× PBS buffer (137 mM NaCl, 2.7 mM KCl, 10 mM Na_2_HPO_4_, and 1.8 mM KH_2_PO_4_) to OD_600_ of 1.0 and placed on ice. About 15 min prior to imaging, cell membrane stain FM 4-64 (5 mM) was added and 2–3 µl of cells were sandwiched between a 24 × 50 mM, no. 1.5 coverslip (Azer Scientific) and a 1.5% agarose pad. All cells were imaged at room temperature with a motorized inverted Nikon Ti-eclipse N-STORM microscope equipped with a 100× objective and ORCA Flash 4.0 digital CMOS C13440 (Hamatsu). Imaging was performed using NIS-Elements software with the microscope in epifluorescence mode. Cells were first imaged in the brightfield (4.5 V, 100 ms exposure). Visualization of the cell membranes was performed in the DsRed channel to ensure the focusing (4.5 V, 50 ms exposure) and then MuGam-GFP was imaged in the GFP channel (4.5 V, 50 ms exposure). Growth, preparation, and imaging were performed for each strain in biological triplicate.

Analysis of cell features was performed with Fiji software (ImageJ) equipped with plugins as described previously: Single-Molecule Biophysics (https://github.com/SingleMolecule/smb-plugins) and MicrobeJ (Ducret *et al.* 2016). Briefly, the nd2 raw images for each strain (4–8 per replicate with a maximum difference of 2 images within triplicate) were concatenated together by channels. The image processing of each channel was carried out the same way and uniformly throughout the field of view. The scale of all images was corrected to fit the Hamamatsu camera scale. The brightfield and DsRed image stacks were auto-scaled while the GFP images were processed with discoidal averaging of 1–5 and intensity scale set at 0–300. Both brightfield and DsRed channels were cleaned by running a Bandpass filter 10_2 with autoscale 5, a rolling sliding stack of 10, and an enhance contrast of 0.1. Channel stacks were converted to 8 bits before analysis in MicrobeJ. For the analysis, hyperstacks combining only the FM 4-64 and GFP channels were generated in MicrobeJ. From these hyperstacks, cell outlines were detected in the DsRed channel using the default method with a threshold of +25. Within identified cells, GFP foci were detected using the maxima features as foci with a Gaussian fit constraint. The exact setup used to identify bacteria and MuGam-GFP foci in MicrobeJ is available (Final Bacteria setup 1_5 foci 90) as a .xml file. After automatic detection, cells were manually sorted to remove poorly fitting outlines or outlines fitting to cells out of focus. Cell features analysis acquired with MicrobeJ (cell ID, cell length, number of foci per cell, foci intensity, and size) was exported as .csv files. Plots and statistical analysis were generated and performed with GraphPad Prism software. At least 650 single cells were analyzed for each condition. Fluorescence and brightfield microscopy data/analysis is included in Supplementary File 3.

## Results

### *Tn*-seq identifies genetic interactions in Δ*priB*, *priC::kan*, and *priA300* strains

DNA replication restart functionally integrates with other processes in *E. coli*. However, experiments to probe this integration have been limited to candidate genetic and biochemical studies. To systematically map connections between DNA replication restart and other processes, we performed *Tn*-seq screens to assess the tolerance of gene disruption in mutant strains restricted to specific pathways of DNA replication restart. Deleting *priB* inactivates the PriA/PriB pathway, the *priA300* allele (which produces an ATPase- and helicase-deficient PriA variant) disables the PriA/PriC pathway, and a *priC*-null mutation (*priC::kan*) inactivates the PriA/PriC and PriC/Rep pathways (Fig. 1) (Sandler 2000; Sandler *et al.* 2001; Windgassen, Wessel, *et al.* 2018; Sandler *et al.* 2021). We therefore carried out screens in each of these backgrounds to independently identify genes with enhanced importance in each genetic background.

Isogenic wild-type (*wt*), Δ*priB*, *priA300*, or *priC::kan E. coli* strains were constructed with the *sulB103* mutation, which encodes an FtsZ variant resistant to SulA-mediated cell division inhibition and bolsters the viability of DNA replication restart mutants (Bi and Lutkenhaus 1990; McCool, Long, *et al.* 2004). Three biological replicate Tn5 transposon libraries with ∼165,000 transposon-insertion mutants were generated for each strain to yield ∼500,000 total insertion mutants in each genetic background. Viable transposon-insertion mutants were selected by plating on SOB solid medium supplemented with trimethoprim (ensuring Tn5 insertion). After pooling to assemble each individual replicate, the libraries were subjected to overnight growth in LB liquid medium forcing direct competition among transposon-insertion mutants. Successive replication initiation events launch prior to cell division in cells grown in rich media, resulting in more than two replication forks on each chromosome (Withers and Bernander 1998; Fossum *et al.* 2007; Hill *et al.* 2012). As a result, the *Tn*-seq screen selected for mutants that allow growth under normal DNA repair and replication restart levels in each of the test strains. Following growth in LB, genomic DNA was isolated from each replicate and prepared for next-generation sequencing. The resulting sequencing data revealed the location of transposon insertions as well as relative transposon-insertion mutant abundance. Each gene in our analysis was assigned a normalized weighted read ratio based on insertion tolerance in the mutant strain compared to the *wt* strain (Burger *et al.* 2017). Positive or negative weighted read ratios reflect gene disruptions that were tolerated better or worse, respectively, in the *wt* strain compared to the mutant strain. Genes with few or no insertions were considered important for growth, and such profiles within the *wt* control strain implicated genes as being essential under the tested growth conditions. By comparing insertion profiles of the *wt* and mutant strains, several genes that were conditionally important in replication restart mutant strains were identified.

**Fig. 2.**
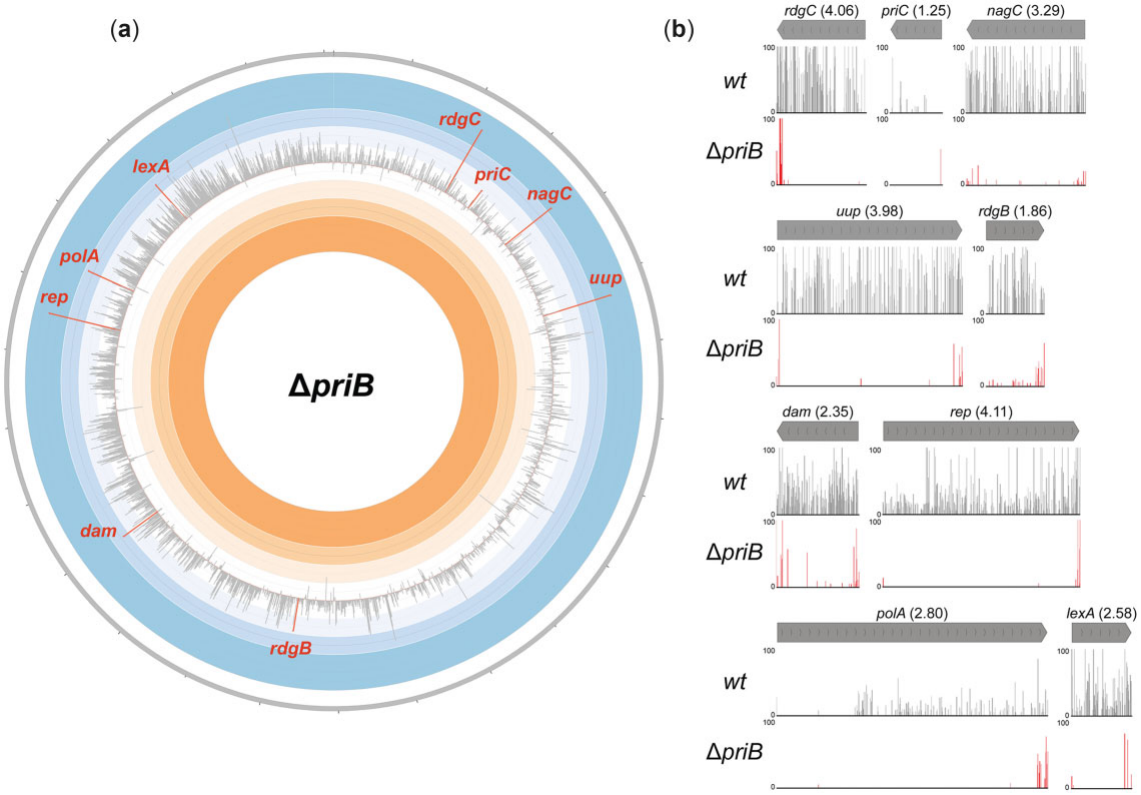
Tn-seq results in Δ*priB E. coli*. a) Circos plot depicting the results of the *Tn*-seq screen in Δ*priB* cells. The effect of single-gene disruption via transposon-insertion was determined by comparing *Tn*-seq read profiles in *wt* vs Δ*priB* conditions, yielding a weighted read ratio. Each bar in the Circos plot represents the weighted read ratio (log_10_) of a single gene where extension into the blue or orange region corresponds to a detrimental or beneficial, respectively, effect of gene disruption. Genes with fewer than three unique transposon-insertions per replicate in the *wt* condition are omitted. The individual disruption of many genes involved in genome maintenance produced some of the most prominent defects and were the focus of the study. Bars for notable genes (*rdgC*, *priC*, *nagC*, *uup*, *rdgB*, *dam*, *rep*, *polA*, and *lexA*) are highlighted. b) MochiView plots for genes highlighted in (a) comparing transposon-insertion locations and read abundance. The corresponding weighted read ratio for each gene is included in parentheses. The maximum read height displayed is 100.

*Tn-*seq data identified several genes as conditionally important in *E. coli* cells lacking the PriA/PriB restart pathway (Δ*priB*). Genes with the strongest *priB* genetic interactions evidenced by weighted read ratios (Fig. 2) and unique insertions (Supplementary Fig. 1a) were selected for subsequent study, except for *rplI* because of its inclusion in the same operon as *priB*. Corroborating previous studies, the screen implicated *rep* (log_10_ weighted read ratio = 4.11) and *dam* (2.35) as genetic interactors with *priB* (Sandler 2000; Boonsombat *et al.* 2006). *priC* (1.25) was a less prominent hit than would be expected given its known synthetic relationship with *priB* (Sandler 2000)*.* However, the modest weighted read ratio for *priC* was due to the limited number of transposon insertion in the *wt* control strain—the *priC* gene tolerated no transposon insertions in the Δ*priB* strain. The expected lethality of a Δ*priB* Δ*priC* double deletion strain was later confirmed. In addition to known genetic interactions, bioinformatic analysis and manual curation of the *Tn*-seq data implicated a variety of novel genes as genetic interactors with *priB*: *rdgC* (4.06), *nagC* (3.29), *uup* (3.98), *rdgB* (1.86), *polA* (2.80), and *lexA* (2.58) (Fig. 2). These top hits (apart from *nagC*) have noted roles in genome maintenance but have not been genetically linked to *priB* prior to this study (d'Ari 1985; Savic *et al.* 1990; Plumbridge 2001; Bradshaw and Kuzminov 2003; Drees *et al.* 2006; Murat *et al.* 2006; Pennetier *et al.* 2008; Romero, Armstrong, *et al.* 2020). The abundance of conditionally important genes in Δ*priB* cells is consistent with PriA/PriB serving as the primary DNA replication restart pathway in *E. coli* (Flores *et al.* 2002).

Disparities in the transposon-insertion profiles between the *wt* control and *priC::kan* or *priA300* mutant strains were relatively modest, resulting in smaller overall weighted read ratios for genes (Supplementary Fig. 1, b and c). This likely was due to basal stress levels being tolerated in both mutant strains since each retained the PriA/PriB pathway (Sandler 2000; Sandler *et al.* 2001, 2021; Windgassen, Wessel, *et al.* 2018). One exception was the clear underrepresentation of transposon insertions in *rep* (4.11) within the *priA300* strain (Supplementary Fig. 1c). This result is consistent with the previously described conditional importance of *rep* in *priA300* cells (Sandler 2000; Mahdi *et al.* 2006; Michel and Sandler 2017). No other genes were identified with significantly different insertion profiles with respect to weighted read ratios in either the *priC::kan* or *priA300* strains relative to the *wt* control (Supplementary Fig. 1, b and c). Interestingly, *priB* had a lower than anticipated weighted read ratio in the *priC::kan* screen, but this was due to a very small number of transposon insertions within *priB* for all strains. This is consistent with a prior observation that the *E. coli priB* gene receives fewer insertions in transposition screens than would be predicted for a gene of its size, which may be due to polar effects on the essential *rpsR* gene and/or *rplI* directly downstream of *priB* within the same operon (Goodall *et al.* 2018).

### Mutations in *priC*, *rep*, *lexA*, *dam*, *rdgC*, *uup*, *nagC*, or *rdgB* confer a dependence on *priB*

**Fig. 3.**
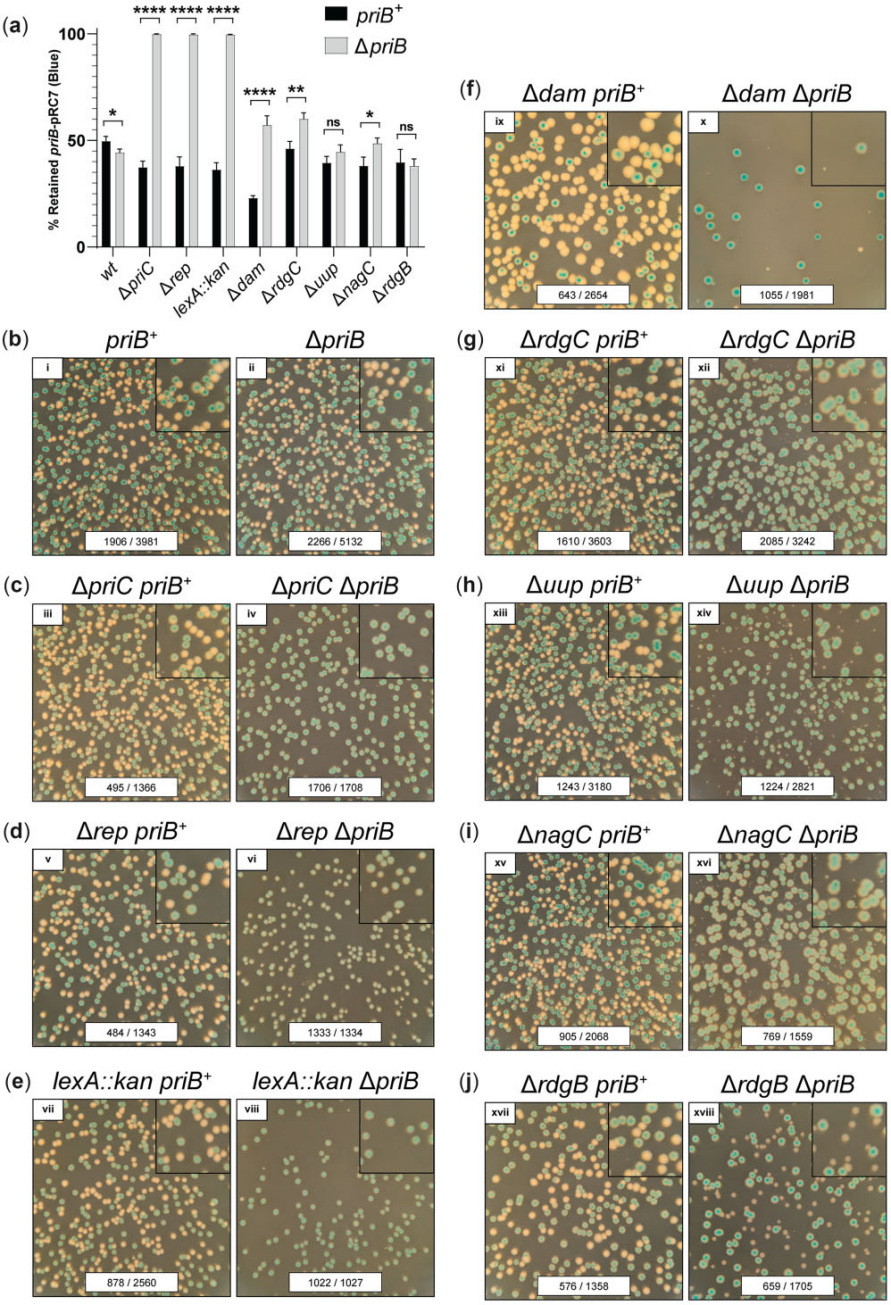
Importance of specific genes in Δ*priB E. coli*. Genes implicated as conditionally important or essential in the Δ*priB Tn*-seq screen were tested with a plasmid retention assay. a) Percentages of colonies that retained *priB*-pRC7 plasmid are shown. Mean values are depicted with error bars representing standard error of the mean. Statistical significance (unpaired student *t*-test) for each strain pair is displayed: *P* < 0.05 (*), *P* < 0.01 (**), and *P* < 0.0001 (****). Representative images from *priB*-pRC7 assay plates are shown as follows: (b) *wt*, (c) Δ*priC*, (d) Δ*rep*, (e) *lexA::kan*, (f) Δ*dam*, (g) Δ*rdgC*, (h) Δ*uup*, (i) Δ*nagC*, and (j) Δ*rdgB*. Each plate image includes raw colony counts for each condition (# of blue colonies/# of total colonies). To better visualize small white colonies, 2.25× magnified insets are included in the upper right-hand corner for each plate image. Each plate was incubated at 37°C for 16 h.

Given the importance of the PriA/PriB pathway as reflected by the Δ*priB Tn*-seq screen results, the remainder of our study interrogated the relationship between *priB* and its genetic interactors. A plasmid retention assay was first used to measure the impact of mutations in genes identified in our *Tn*-seq screen on cell viability with or without chromosomal *priB* (Bernhardt and de Boer 2004; Romero, Chen, *et al.* 2020). The assay followed retention of an unstable, low-copy plasmid (*priB*-pRC7, which contained *priB* and the *lac* operon) in *priB*^+^ or Δ*priB* strains with chromosomal deletions of the *lac* operon and genes identified as conditionally important in the Δ*priB Tn*-seq screen. Plasmid retention or loss was marked by colony color (blue or white, respectively) when plated on SOB-agar containing X-gal and IPTG (Fig. 3). Importantly, these *priB-pRC7* retention experiments did not rely on constructing double mutant strains, which are particularly susceptible to suppressor mutations during liquid growth experiments (Sandler 2000). Instead, the strains were tested in a restricted experimental window following *priB*-pRC7 plasmid loss (immediately prior to plating).

In line with previous genetic results, deletion of *priC* or *rep* in Δ*priB* cells resulted in persistent retention of *priB*-pRC7, strongly supporting their known synthetic lethal relationships with *priB* (Fig. 3, c and d) (Sandler 2000). Screening of a newly identified genetic interaction revealed that *lexA* and *priB* also form a synthetic lethal pair in our genetic background (Fig. 3e). LexA is a transcriptional repressor that undergoes auto-proteolysis to induce the SOS DNA-damage response genes (d'Ari 1985; Giese *et al.* 2008). As a result, disruption of *lexA* causes constitutive SOS expression, and it follows that induction of one or more SOS genes is toxic to Δ*priB* cells (McCool, Long, *et al.* 2004). For mutations in *priC*, *rep*, or *lexA*, the extent of plasmid loss was equivalent to control levels in *priB*^+^ cells (Fig. 3, a–e).

In contrast to the robust and consistent *priB*-pRC7 retention characteristics of the mutant strains described above, mutations in *dam*, *rdgC*, *uup*, *nagC*, or *rdgB* did not entirely prevent plasmid loss when paired with a *priB* deletion (Fig. 3, f–j). However, compared to plasmid-containing blue colonies, white colonies (lacking *priB*-pRC7) formed by these double mutants were smaller, indicative of reduced growth rates. For Δ*rdgB* Δ*priB*, the disparity in size between blue and white colonies was modest (Fig. 3J). However, when other gene deletions (Δ*dam*, Δ*rdgC*, Δ*uup*, or Δ*nagC*) were paired with Δ*priB*, the resulting plasmid-less white colonies were particularly small and difficult to quantify (Fig. 3, f–i). As a result, disparities in colony size for many of these strains is likely a better proxy of cellular health than a plasmid retention percentage (Fig. 3a).

Previous studies have noted a synthetic lethal relationship between *dam* and *priB*, suggesting that DSBs accumulating in Δ*dam* cells are preferentially funneled into the PriA/PriB pathway for restart following repair (Boonsombat *et al.* 2006). While our data do not confirm a synthetic lethal relationship between *dam* and *priB*, our *priB*-pRC7 retention results identify a strong conditional importance of *dam* in Δ*priB* cells based on a disparity in colony size (Fig. 3f). The decreased growth rate of white colonies evident in the assay also confirmed the conditional importance of *rdgC*, *uup*, *nagC*, and *rdgB* in Δ*priB* cells (Fig. 3, g–j). While RdgC (an inhibitor of RecA recombinase activity), Uup (a branched DNA intermediate binding protein), and RdgB (a noncanonical purine pyrophosphatase) have been implicated in genome maintenance processes, these results now map the genes’ interactions with the PriA/PriB restart pathway (Bradshaw and Kuzminov 2003; Moore *et al.* 2003; Drees *et al.* 2006; Murat *et al.* 2006; Romero, Armstrong, *et al.* 2020; Romero, Chen, *et al.* 2020). Surprisingly, conditional importance in Δ*priB* cells extended to *nagC*, which encodes a transcriptional repressor that coordinates *N*-acetylglucosamine biosynthesis but has no known role in DNA metabolism (Plumbridge 2001; Pennetier *et al.* 2008). These data support the notion of conditional importance for *dam*, *rdgC*, *uup*, *nagC*, or *rdgB* in Δ*priB* cells (Fig. 3, f–j).

### Disruption of *rdgB* in a Δ*priB* strain confers a fitness defect

**Fig. 4.**
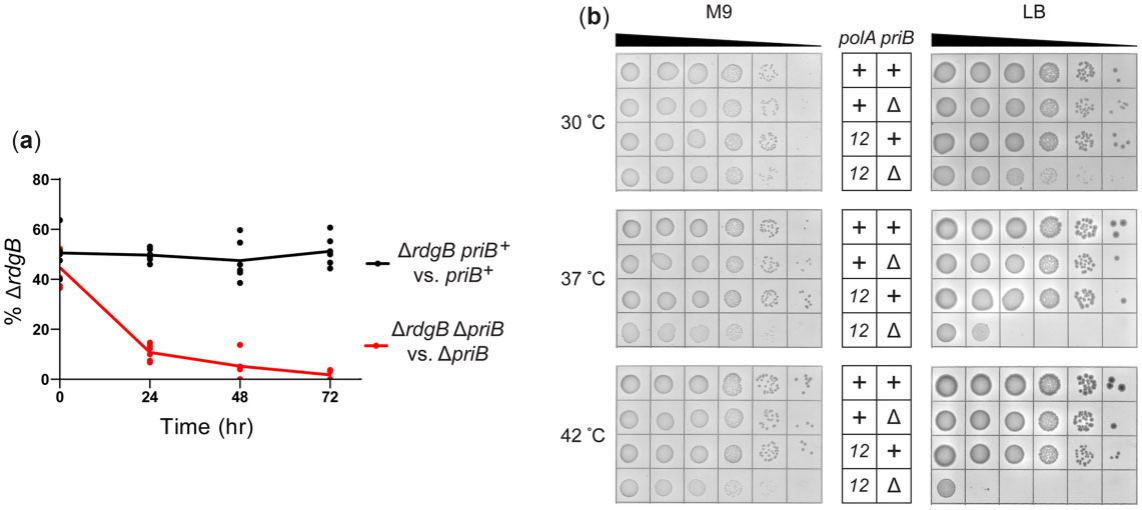
Importance of *rdgB* and Pol I polymerase activity in Δ*priB E. coli*. a) Growth competition examining the effect of a *rdgB* mutation on fitness *for priB*^+^ or Δ*priB* strains. Trendlines for each series intersect the mean, and biological triplicate data points are presented for competitions done in duplicate. b) Effect of the *polA12(ts)* allele on *priB*^+^ or Δ*priB* strains. Strains were spot plated on minimal (M9, left) or rich (LB, right) media and incubated at 30, 37, or 42°C. Dilutions (from left to right) are 10× serial dilutions from normalized overnight cultures.

The disparity of colony sizes in the *priB*-pRC7 retention assay provided only moderate evidence that *rdgB* is conditionally important in Δ*priB* cells. To examine the *rdgB priB* genetic relationship more confidently, the fitness of strains combining *rdgB* and *priB* mutations was tested in a growth competition assay. In this assay, the effect of a *rdgB* deletion was examined within a *priB*^+^ competition (*priB*^+^ vs Δ*rdgB priB*^+^) and within a Δ*priB* competition (Δ*priB* vs Δ*rdgB* Δ*priB*). A synthetic fitness defect would result in selective loss of Δ*rdgB* Δ*priB* in the latter competition. A reporter mutation (Δ*araBAD*) in one strain of each competition was utilized to quantify the relative Δ*rdgB* abundance throughout each competition. As expected for a synthetic *rdgB priB* relationship, simultaneous deletion of both genes caused a pronounced fitness defect within 24 hours when grown in competition with *rdgB*^+^ Δ*priB* cells (Fig. 4a, red). In contrast, Δ*rdgB priB*^+^ cells exhibited no detectable fitness defect when grown in competition with *wt* cells, as evidenced by steady relative abundance within the *priB*^+^ competition (Fig. 4a, black). These results confirm that *rdgB* is not essential in a Δ*priB* strain, but that it is conditionally important. The mild defect in growth rate of Δ*rdgB* Δ*priB* colonies (Fig. 3j) but clear fitness defect (Fig. 4a) align well with *rdgB* as a relatively weak hit from our Δ*priB Tn*-seq screen (Fig. 2 and Supplementary Fig. 1a).

### Polymerase I activity is conditionally important for Δ*priB* cells

The *Tn*-seq screen suggested a genetic relationship between *priB* and *polA* (Fig. 2). However, the essential nature of *polA* ruled out simple gene deletion experiments to further examine this link (Joyce and Grindley 1984; Joyce *et al.* 1985). Inspection of the transposon-insertion profiles (Fig. 2b) suggests that only certain regions of *polA* are conditionally important for survival in Δ*priB* cells. Specifically, regions of the gene that encode the C-terminal 3′-5′ exonuclease and polymerase domains of DNA polymerase I (Pol I) poorly tolerated transposon insertions in the Δ*priB* strain compared to the *wt* strain. These two domains comprise the Klenow fragment of Pol I (Klenow and Henningsen 1970). Conversely, the portion of *polA* encoding the 5′–3′ exonuclease domain poorly tolerated insertions in both the Δ*priB* and *wt* strains, consistent with this domain encoding the essential function of *polA* in rich media (Joyce and Grindley 1984).

To test the importance of the polymerase activity of Pol I in Δ*priB* cells, we utilized the *polA12(ts)* mutant allele. This mutation encodes a Pol I variant with severely inhibited polymerase activity at high temperatures (Lehman and Chien 1973; Uyemura and Lehman 1976; Camps and Loeb 2005). In addition, *polA12(ts)* is synthetically lethal with a *priA* mutation under nonpermissive conditions (Lee and Kornberg 1991; Kogoma 1997). Spot plate assays examined the viability of *polA12(ts)* Δ*priB* and control strains at increasing temperatures on LB (rich) or M9 (minimal) media to determine the conditional importance of Pol I polymerase activity (Fig. 4b). In agreement with the *Tn*-seq screen results, *polA12(ts)* Δ*priB* cells displayed temperature-sensitive synthetic defects on LB media. At 37°C, the double mutant was at least 100x less viable than the *polA12(ts) priB^+^* strain, and this effect was exacerbated to ∼1,000x at 42°C. The *polA12(ts)* mutation appeared to cause a reduced growth rate of Δ*priB* cells even at 30°C, evidenced by the smaller colony sizes in the double mutant. Based on previous studies, this detrimental effect is likely driven by reduced polymerase activity (Lehman and Chien 1973; Uyemura and Lehman 1976; Camps and Loeb 2005). Interestingly, *polA12(ts)* Δ*priB* strain viability was significantly restored by plating on M9 (minimal) media. This partial suppression likely stems from fewer concurrent rounds of DNA replication initiation in minimal media, and it underpins the importance of efficient genome maintenance in nutrient-rich environments (Withers and Bernander 1998; Fossum *et al.* 2007; Hill *et al.* 2012).

### Mutations in *rep*, *lexA*, *polA*, or *dam* cause sensitivity to exogenous DSBs

**Fig. 5.**
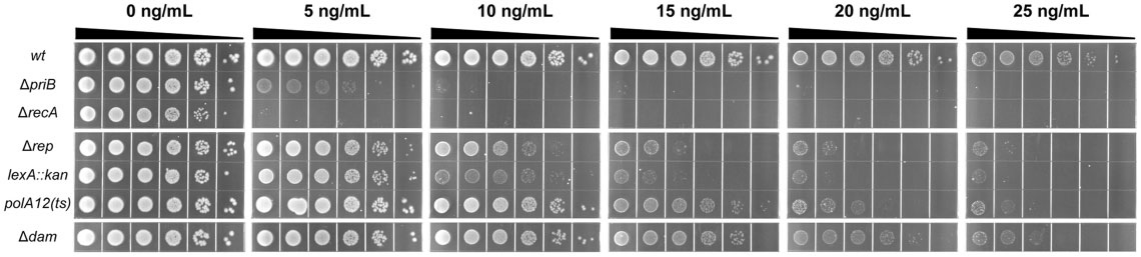
Effects of *priB*, *rep*, *lexA*, *polA*, or *dam* mutations on DNA-damage sensitivity in *E. coli*. Sensitivity of mutants to DSBs was examined by spot plating on LB agar with 0–25 ng/ml ciprofloxacin. A *recA* deletion strain was utilized as a positive control of ciprofloxacin hypersensitivity. Dilutions (from left to right) are 10× serial dilutions from normalized overnight culture. Displayed spot plate data are representative of three replicates.

A prior study demonstrated a synthetic lethal relationship between *priB* and *dam* and suggested that this relationship may result from DSBs formed in *dam* mutants being funneled into the PriA/PriB restart pathway following their repair (Boonsombat *et al.* 2006). Therefore, we examined whether other genes identified in the Δ*priB Tn*-seq screen could be driving toxicity through enhanced DNA-damage accumulation. Mutant strains were spot plated onto LB supplemented with sublethal concentrations of the DSB-inducing antibiotic ciprofloxacin (Fig. 5) (Willmott and Maxwell 1993; Tamayo *et al.* 2009). A *recA* deletion strain was utilized as a positive control for hypersensitivity (Klitgaard *et al.* 2018) and was inviable at 5 ng/ml ciprofloxacin. Notably, a Δ*priB* strain also exhibited extreme hypersensitivity and was inviable at 10 ng/ml ciprofloxacin. Similar sensitivity was reported recently for Δ*priB E. coli* (Mallikarjun and Gowrishankar 2022). Mutations in *rep* and *lexA* led to viability defects at 10 ng/ml ciprofloxacin but were significantly more resistant than Δ*priB* or Δ*recA* strains. At 15 ng/ml ciprofloxacin, the Δ*dam* and *polA12(ts)* mutants began to display defects as well. We note that the reduced growth rate of *dam* mutants in the presence of DNA-damaging agents has been linked to a reduction in replication initiation, which may be leading to smaller colony sizes with inhibitory ciprofloxacin concentrations (Sutera and Lovett 2006). Other mutants identified in the Δ*priB Tn*-seq screen were not sensitized to ciprofloxacin (Supplementary Fig. 2). These results suggest cellular roles for *priB*, *recA*, *dam*, *rep*, *lexA*, and *polA* in prevention and/or repair of DNA damage in vivo.

### Visualizing DSBs in vivo with MuGam-GFP

Sensitization of *rep*, *lexA*, *polA*, and *dam* mutants to ciprofloxacin suggests that these mutant strains may also have enhanced levels of endogenous DSBs. To test this hypothesis, mutations were transduced into an *E. coli* strain (SMR14334) encoding inducible MuGam-GFP, a DSB sensor protein, and the extent of DSB accumulation was determined in vivo with fluorescence microscopy (Supplementary Fig. 3, a, b, and d) (Shee *et al.* 2013).

**Fig. 6.**
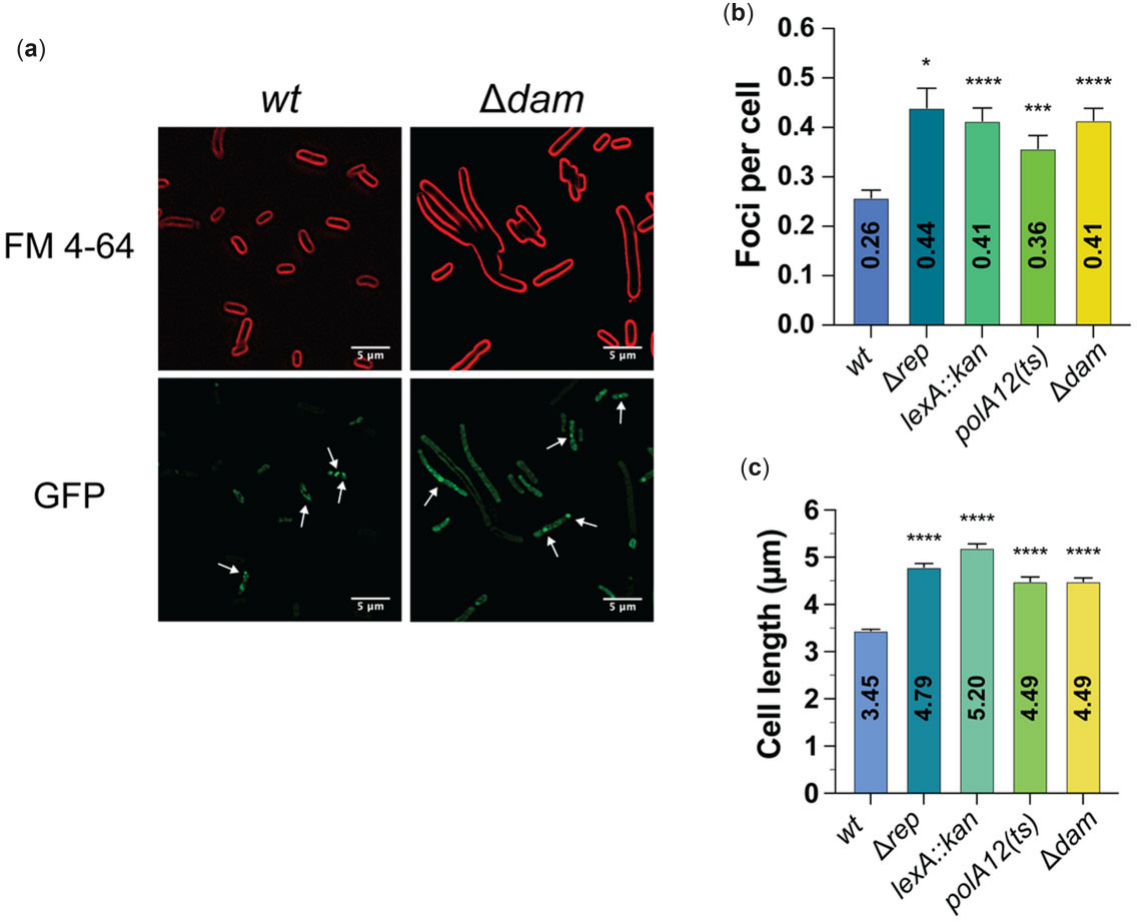
Enhanced DSB formation in mutant *E. coli* strains. a) Representative images depicting MuGam-GFP foci and FM 4-64-stained membranes for *wt* (left) and Δ*dam* (right) strains. The abundance of MuGam-GFP foci per cell (b) and measured cell lengths (c) are displayed for *wt*, Δ*rep*, *lexA::kan*, *polA12(ts)*, and Δ*dam* strains. b, c) Mean values are depicted with error bars representing standard error of the mean. Statistical significance (*U* Mann–Whitney) for each strain compared to the *wt* control is displayed: *P* < 0.05 (*), *P* < 0.001 (***), and *P* < 0.0001 (****).

MuGam-GFP foci were more abundant in a *dam* deletion strain than in the *wt* strain (Fig. 6, a and b) (Nowosielska and Marinus 2005). These mutant cells were also severely filamented which is a hallmark of DNA damage in *E. coli* (Fig. 6, a and c) (Huisman and d'Ari 1981). Consistent with their sensitivity to ciprofloxacin (Fig. 5), mutations in *rep*, *lexA*, or *polA* also resulted in increased MuGam-GFP focus formation (Fig. 6b and Supplementary Fig. 3, a, b, and d) and cell length (Fig. 6c and Supplementary Fig. 3c). Notably, a *rdgC* mutant displayed significant accumulation of DSBs (Supplementary Fig. 3, a, b, and d) while exhibiting only a moderate increase in cell length (Supplementary Fig. 3c) and no observable sensitization to ciprofloxacin (Supplementary Fig. 2).

The evidence of DSB accumulation and cell filamentation in other mutants tested is less compelling. Mutations in *priC*, *uup*, or *rdgB* produce only mild filamentation phenotypes, and there was limited evidence that disrupting *priC* enhances DSB levels (Supplementary Fig. 3, a–d). In fact, *nagC* and *rdgB* mutant strains exhibited significantly lower abundance of MuGam-GFP foci compared to the *wt* control and GFP focus levels in the *nagC* mutant approached the lower limit of detection. For the *nagC* mutant, this may have been caused by a significantly lower level of mean fluorescence (Supplementary Fig. 3e).

### Modulating RecA function partially suppresses *lexA* or *rdgC* mutational effects on Δ*priB* cells

Mutations in *dam*, *rep*, *lexA*, *polA*, or *rdgC* increase DSB formation in vivo (Fig. 6, a and b and Supplementary Fig. 3, a, b, and d). In most cases, this effect is accompanied by sensitization to ciprofloxacin (Fig. 5 and Supplementary Fig. 2) and cell filamentation (Fig. 6c and Supplementary Fig. 3, a and c). Deleting *dam* or hindering Pol I polymerase activity can cause persistent ssDNA gaps that form DSBs when subsequent replisomes collide (Glickman 1975; Cao and Kogoma 1995; Mojas *et al.* 2007; Michel *et al.* 2018). Similarly, a loss of Rep accessory helicase activity correlates with more stalled replication forks that can create DSBs when they are encountered by subsequent replisomes (Michel *et al.* 1997; Seigneur *et al.* 1998; Michel *et al.* 2018). Our data strongly suggest an increase in DSB formation in *lexA* or *rdgC* mutants, which likely accounts for their genetic relationships with *priB*, but their mode of DSB formation is less clear.

Previous work has shown that loss of PriA or Rep helicase activity at stalled replication forks can cause inappropriate RecA recombinase loading mediated by the ssDNA gap repair proteins RecFOR (Mahdi *et al.* 2006). After it is loaded by RecFOR, RecA is hypothesized to reverse a stalled replication fork to form a Holliday junction, also known as a “chicken-foot” structure (Robu *et al.* 2001; Courcelle *et al.* 2003). Because LexA or RdgC inhibit the activity of cellular RecA [via transcriptional repression (d'Ari 1985) or physical inhibition (Drees *et al.* 2006), respectively], we hypothesized that more stalled forks were reversed in *lexA* or *rdgC* mutants. The DSBs observed in vivo (Fig. 6b and Supplementary Fig. 3, a and b) could form in these mutants when the “chicken-foot” structures were encountered by additional replisomes (from multifork replication conditions in rich media) or upon processing by RuvABC, the Holliday junction resolvase (Seigneur *et al.* 1998, 2000; Withers and Bernander 1998; Michel *et al.* 2018).

**Fig. 7.**
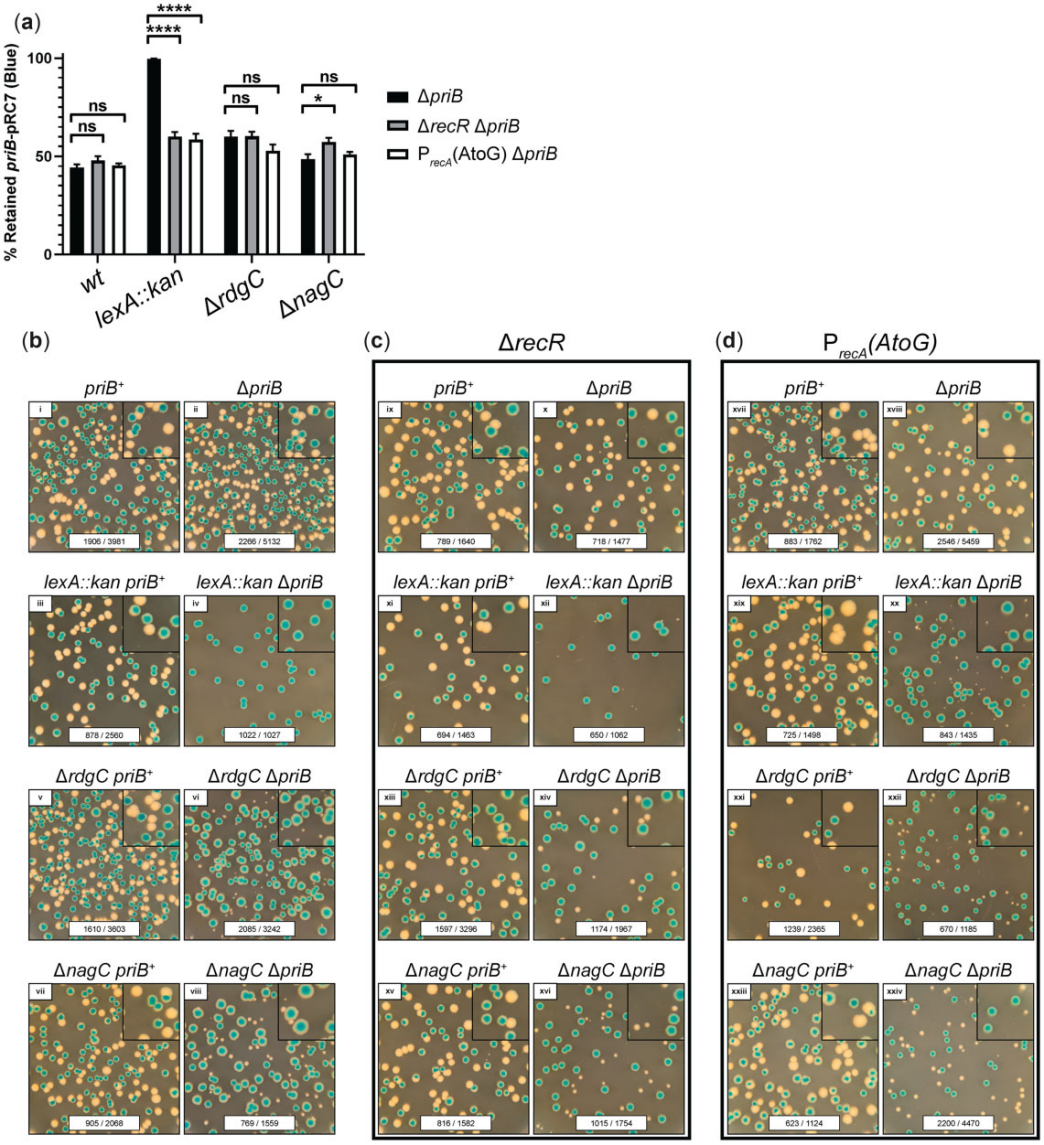
Modulating RecA activity partially suppresses mutational effects on Δ*priB E. coli*. a) Plasmid (*priB*-pRC7) retention in Δ*priB* strains is shown along with strains also carrying a *recR* deletion or *recA* promoter mutation. Mean values are depicted with error bars representing standard error of the mean. Statistical significance (unpaired Student’s *t*-test) for each strain pair is displayed: *P* < 0.05 (*) and *P* < 0.0001 (****). b) Representative images of *priB*-pRC7 assay plates are presented for *wt*, *lexA::kan*, Δ*rdgC*, and Δ*nagC* strains with or without chromosomal *priB*. This experiment was extended to strains with (c) a *recR* deletion or (d) a mutation in *recA*’s promoter. b–d) Each image includes raw colony counts for each condition (# of blue colonies/# of total colonies). To better visualize small white colonies, 2.25× magnified insets are included in the upper right-hand corner. Each plate was incubated at 37°C for 22 h.

To test this hypothesis, we examined the effect of RecA modulation on *lexA* or *rdgC* mutants in the *priB*-pRC7 plasmid retention assay (Fig. 7). Previously, our results identified a conditional essentiality of *lexA* in Δ*priB* cells based on robust retention of the *priB*-pRC7 plasmid (Figs. 3e and 7, a and b). After deleting *recR* in this strain (inactivating the RecFOR pathway), we observed viable *lexA::kan* Δ*priB* white colonies (Fig. 7c). The resulting colonies were quite small, consistent with growth defects, but these results strongly support a partial suppression of *lexA::kan* Δ*priB* via *recR* deletion. Likewise, the conditional importance of *rdgC* in Δ*priB* cells (Fig. 3g) was partially suppressed with a *recR* deletion, evidenced by significantly larger plasmid-less white colonies (Fig. 7c).

In addition to restricting the scope of RecA activity in vivo with a *recR* deletion, we hypothesized that reducing the cellular levels of RecA would also produce a suppressive effect. To accomplish this, we utilized a *recA* promoter mutation, P_*recA*_(AtoG), which decreases *recA* expression (Weisemann and Weinstock 1985; 1991; Romero, Chen, *et al.* 2020). This mutation also suppressed the effects of *lexA* or *rdgC* mutations in Δ*priB* cells, and the degree of suppression was strikingly similar to that of a *recR* deletion (Fig. 7d). To rule out general suppression ability of these RecA modulations, we tested their effect on other mutants identified in our *Tn*-seq screen. We only observed modest evidence of suppression by RecA modulation in Δ*nagC* Δ*priB* strains when comparing the relative sizes of white and blue colonies (Fig. 7, b–d). Taken together, these results suggest that *lexA* or *rdgC* deletions promote inappropriate and/or excessive RecA activity causing stalled replication forks to physically reverse and eventually devolve to DSBs upon replisome collision or Holliday junction processing.

## Discussion

DNA replication restart reactivates prematurely abandoned DNA replication sites that have failed due to replisome encounters with damaged DNA or proteins tightly bound to chromosomes. Our knowledge of the coordination between DNA replication restart and other genome maintenance pathways has been limited by a lack of systematic genetic studies assessing the importance of genes to each replication restart pathway in *E. coli.* To determine links between replication restart and other cellular processes, we have identified genes that are conditionally essential or important in *E. coli* strains with inactivated replication restart pathways. High-density transposon mutant libraries in strains lacking *priB*, *priC*, or with the *priA300* mutation were analyzed after growth on rich media. These mutations inactivate the PriA/PriB, PriC/Rep and PriA/PriC, or PriA/PriC pathways, respectively (Fig. 1) (Sandler 2000). Comparison of transposon-insertion profiles to a *wt* control strain revealed genetic interactions with specific replication restart pathways. It is possible these replication restart mutations elicit other cellular effects such as perturbations in gene expression levels, and these off-target impacts may affect cellular function. Future transcriptome experiments will help determine if the effects of these mutations are restricted to DNA replication restart pathway accessibility.

Several genes were found to be conditionally essential or important in Δ*priB E. coli*, which specifically lacks the PriA/PriB pathway (Fig. 2 and Supplementary Fig. 1a). In contrast, only one gene (*rep*) displayed significant importance in *priA300 E. coli* and no genes were significantly conditionally important in *priC::kan E. coli* (Supplementary Fig. 1, b and c). These results point to PriA/PriB serving as the major replication restart pathway integrated within the larger genome maintenance program in *E. coli*, consistent with prior data (Flores *et al.* 2002). It is possible that the PriA/PriC and PriC/Rep pathways operate on DNA replication fork substrates that are rarely generated under the conditions tested in our experiments (Heller and Marians 2005a). It is also possible that the PriA/PriB pathway can compensate for the PriA/PriC and PriC/Rep pathways but the latter two pathways cannot compensate for PriA/PriB.

**Fig. 8.**
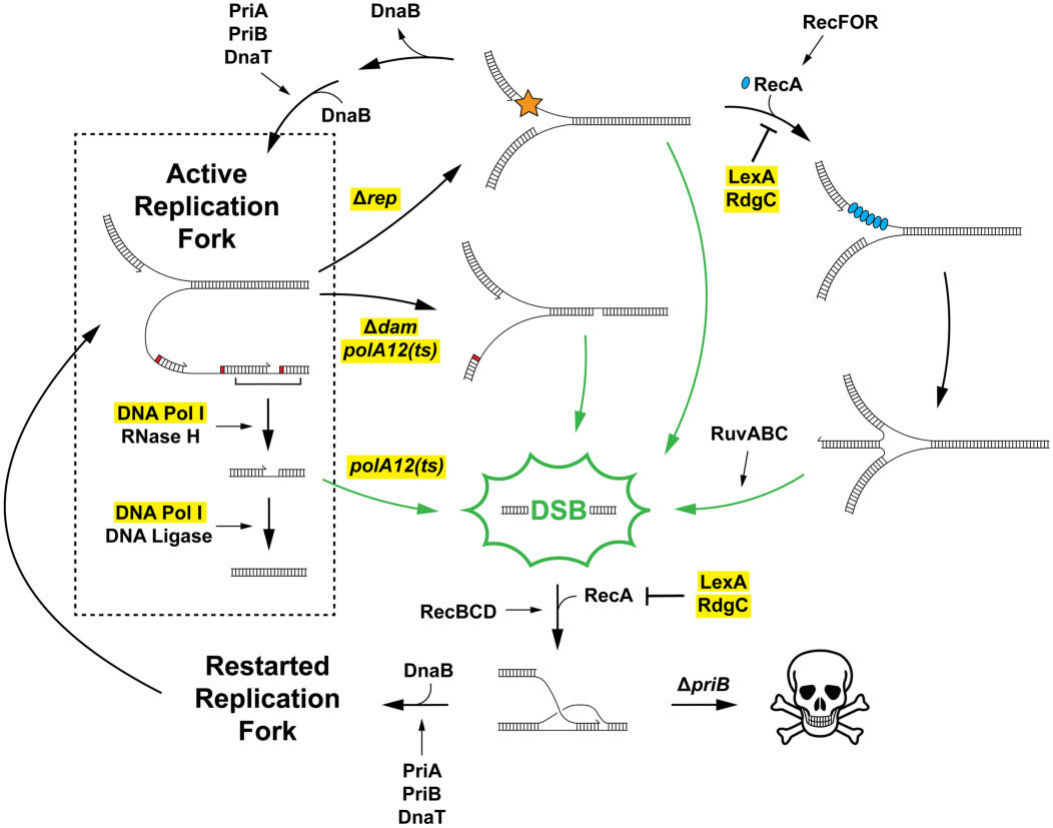
DSBs accumulate from a variety of sources and are funneled into the PriA/PriB replication restart pathway following their repair. An active replication fork facilitates continuous DNA synthesis on the leading strand, while lagging strand synthesis is discontinuous and downstream processing is required by other enzymes. These productive processes are contained within the box. Several damaging paths are also shown. Loss of Rep causes an increase in replication fork collisions with nucleo-protein complexes (star). The most severe collisions cause lethal replisome dissociation unless DNA replication restart is carried out, which is primarily facilitated by the PriA/PriB pathway. Increased mismatch repair (without Dam methylation) or loss of Pol I polymerase activity following DNA repair or during Okazaki fragment maturation cause persistent ssDNA gaps. RecA loading at stalled replication forks mediated by RecFOR can drive fork reversal, which is inhibited by LexA or RdgC. Stalled/reversed replication forks and ssDNA gaps are DSB-prone substrates; if they are not efficiently repaired, they lead to DSBs when they are encountered by subsequent replisomes. When DSBs form, they are recognized and repaired with homologous recombination (RecA is loaded via RecBCD pathway). The resulting D-loop substrate is shuttled into the PriA/PriB pathway to reinitiate DNA replication and maintain cell viability. The genes/proteins examined with targeted genetic analyses in this study are highlighted.

Deletion of *rep* was found to be detrimental in both Δ*priB* and *priA300* strains, consistent with a general importance of the Rep helicase in genome maintenance (Fig. 2 and Supplementary Fig. 1c). Rep can be recruited to stalled replication forks via interaction with PriC where it helps facilitate DNA replication restart in the PriC/Rep pathway (Fig. 1) (Syeda *et al.* 2019; Nguyen *et al.* 2021). PriC interaction with Rep also stimulates its helicase activity (Heller and Marians 2005b). It may be that Δ*priB* and *priA300 E. coli* strains rely more heavily on the PriC/Rep pathway or that deletion of *rep* places a larger burden on the PriA/PriB or PriA/PriC DNA replication pathways. In accordance with the latter possibility, Rep also interacts with the replicative helicase, DnaB, which localizes Rep helicase activity to sites of DNA replication and is thought to enhance its ability to remove tightly associated protein barriers ahead of the replication fork (Syeda *et al.* 2019). The absence of Rep results in increased fork stalling, replisome dissociation, and DSBs if left unrepaired, which could also feed into the PriA/PriB pathway (Fig. 8) (Michel *et al.* 1997, 2018; Seigneur *et al.* 1998).

In addition to the known importance of *rep* in Δ*priB* cells, our results corroborated the importance of *dam* and *priC* in Δ*priB* cells (Fig. 3, c and f) (Sandler 2000; Boonsombat *et al.* 2006). In cells lacking Dam methyltransferase, both DNA strands are nicked and excised at equal frequency by methyl-directed mismatch repair enzymes, causing persistent ssDNA gaps that lead to DSBs when encountered by a replisome (Fig. 8) (Mojas *et al.* 2007; Michel *et al.* 2018). Interestingly, Δ*dam* cells are also associated with chromosomal overreplication, likely stemming from DSB repair feeding into DNA replication restart (Raghunathan *et al.* 2019). Overreplication could exacerbate DSB accumulation in Δ*dam* cells and it may elicit a similar effect in other DSB-causing mutants described in this study. The synthetic lethality of the Δ*priB* Δ*priC* combination was also confirmed (Fig. 3c), although the genetic relationship was not detected in either the Δ*priB* or *priC::kan Tn*-seq screens due to a small number of transpositions insertions mapped for *priB* or *priC* in the *wt* reference strain (Fig. 2 and Supplementary Fig. 1b). This may be due to a transposition recalcitrance for *priC* as has been noted for *priB* (Goodall *et al.* 2018). Thus, it is possible that additional *priB*, *priC*, or *priA300* genetic interactions beyond those described here may exist and that limitations of the *Tn*-seq approach could mask their identification.

The *Tn*-seq results in the Δ*priB* strain and targeted genetic experiments identified a host of novel *priB* genetic interactors: *lexA*, *polA*, *rdgC*, *uup*, *nagC*, and *rdgB* (Figs. 2 and 3). In addition to mutant strains expected to exhibit DSB accumulation (*rep* and *dam*), in vivo measurements detected significant DSB accumulation for *lexA*, *polA*, and *rdgC* mutants (Fig. 6, a and b and Supplementary Fig. 3, a and b). Formation of DSBs in these mutant strains was correlated with longer cell lengths (Fig. 6c and Supplementary Fig. 3c) and sensitization to the DSB-inducing antibiotic ciprofloxacin (Fig. 5 and Supplementary Fig. 2), except for the *rdgC* deletion.

Pol I is known to utilize its polymerase activity to fill ssDNA gaps during Okazaki fragment synthesis and following DNA repair (Lehman and Chien 1973; Glickman 1975; Uyemura and Lehman 1976; Cao and Kogoma 1995). The results shown here suggest this activity is especially important in Δ*priB* cells (Figs. 2b and 4b). We hypothesize that persistent ssDNA gaps are formed in *polA12(ts)* mutant strains at elevated temperatures, which lead to DSBs if left unrepaired when encountered by a replisome (Fig. 8) (Michel *et al.* 2018). This notion is supported by *polA12(ts)* Δ*priB* phenotype suppression on minimal media (Fig. 4b) when multifork DNA replication is less likely to occur and cause DSBs from collisions with ssDNA gaps (Withers and Bernander 1998; Fossum *et al.* 2007; Hill *et al.* 2012).

The formation of DSBs in *lexA* or *rdgC* deletion strains is less straightforward. Previous work has shown that the absence of PriA or Rep helicase activity can allow the RecFOR mediator proteins to inappropriately load RecA at stalled replication forks (Moore *et al.* 2003; Mahdi *et al.* 2006). Upon binding, RecA can physically reverse the stalled fork forming a “chicken-foot” structure (Fig. 8). DSBs will form from these structures when they are encountered by subsequent replication forks or when they are processed by RuvABC (Fig. 8) (Seigneur *et al.* 1998, 2000; Withers and Bernander 1998; Michel *et al.* 2018). Therefore, we hypothesized that the higher levels of DSBs formed in *lexA* or *rdgC* mutants (Fig. 6b and Supplementary Fig. 3, a, b, and d) was caused by excessive RecA activity: either by disrupting its transcriptional repressor (LexA) or by removing a RecA inhibitor (RdgC). Increasing the activity of RecA by disrupting *lexA* or *rdgC* would in turn promote unwarranted RecA activity (Fig. 8). Consistent with this notion, the effects of *lexA* or *rdgC* mutations on Δ*priB* cells were partially suppressed by disabling the RecFOR pathway (with a *recR* deletion) or by inhibiting cellular RecA activity by decreasing its expression with a promoter mutation (P_*recA*_(AtoG)) (Fig. 7). Future experiments are required to probe these relationships further by attempting suppression with deletion of *ruvC* (Fig. 8). Notably, the Δ*recR* and P_*recA*_(AtoG) suppression attempts partially restored the growth rates of Δ*rdgC* Δ*priB* colonies, while permitting (albeit limited) viability of *lexA::kan* Δ*priB* cells. It is likely that the SOS DNA-damage response induces the expression of one or more genes (other than *recA*) that are harmful to Δ*priB* cells.

DSBs can form in a variety of different ways in the cell. Disrupting genes identified in the Δ*priB Tn*-seq screen likely increased DSB levels by promoting the formation of DSB-prone substrates (stalled/reversed replication forks and ssDNA gaps), which are encountered by subsequent replication complexes in rich media (Fig. 8) (Withers and Bernander 1998; Fossum *et al.* 2007; Hill *et al.* 2012; Michel *et al.* 2018). While DSBs are problematic, cells can survive if they are readily recognized and repaired. In *E. coli*, DSB repair is usually carried out by RecBCD, which processes DSBs before loading RecA to catalyze strand invasion and create a D-loop site for DNA replication restart (Fig. 8) (Dillingham and Kowalczykowski 2008). The DSBs formed in *rep*, *lexA*, *polA*, *dam*, and *rdgC* mutants can still be recognized and repaired by the RecBCD pathway to form D-loops, which subsequently undergo DNA replication restart via the PriA/PriB pathway (Heller and Marians 2005a; Boonsombat *et al.* 2006; Sasaki *et al.* 2007; Windgassen, Leroux, *et al.* 2018). We hypothesize that these mutations are synergistic with a *priB* deletion because DSBs are committed to a nonproductive pathway (when *priB* is absent) and stagnant D-loops may ultimately lead to cell death (Fig. 8). Furthermore, while most DSB-causing mutants showed some sensitization to ciprofloxacin, *priB* and *recA* deletion strains exhibited extreme sensitization, with *priB* deletion sensitizing cells just slightly less than a *recA* deletion (Fig. 5). Taken together, our data strengthen the experimental support for a link between DSB repair and the PriA/PriB pathway of DNA replication restart. Our results do not exclude the possibility that the PriA/PriC or PriC/Rep pathways play more minor roles in replication restart after DSB repair as has been recently suggested (Mallikarjun and Gowrishankar 2022).

The results presented here highlight a variety of new questions and exciting opportunities of study. While *uup*, *nagC*, and *rdgB* are conditionally important in Δ*priB* cells, their disruption does not appear to cause DSBs in the conditions tested (Supplementary Fig. 3, a, b, and d). Most puzzling is the genetic relationship between *priB* and *nagC*, a transcriptional repressor that coordinates the biosynthesis of *N*-acetylglucosamine, a component of the bacterial cell wall (Plumbridge 2001; Pennetier *et al.* 2008). Deletion of *nagC* led to an aberrant cell morphology (Supplementary Fig. 3a), which may have caused the mutant’s extremely low level of mean fluorescence in our experiments (Supplementary Fig. 3e). It is possible the perturbed cell membrane morphology is linked to DNA damage, similar to observations made with perturbed nuclear envelopes upon loss of lamin proteins in cancer cells (Denais *et al.* 2016). It is also possible that deletions of *uup*, *nagC*, or *rdgB* directly impact PriC-dependent replication restart, which would result in strong genetic interactions with *priB.* Future studies will be required to further probe these possibilities. Taken together, our findings have better defined a primary role for the PriA/PriB replication restart pathway following DSB repair in *E. coli* and have established important links that integrate replication restart processes into a larger genome maintenance program in bacteria.

## Supplementary Material

jkac295_Supplemental_Material_Legends

jkac295_Supplementary_Figure_S1

jkac295_Supplementary_Figure_S2

jkac295_Supplementary_Figure_S3

jkac295_Supplementary_File_S1

jkac295_Supplementary_File_S2

jkac295_Supplementary_File_S3

jkac295_Supplementary_Table_S1

jkac295_Supplementary_Table_S2

## Data Availability

Raw sequencing data for *Tn*-seq experiments can be found at NCBI SRA under BioProject ID PRJNA837116. All microscopy data can be found at Dryad repository (https://doi.org/10.5061/dryad.547d7wmbx). Supplemental material is available at G3 online.
