# Supplementary material for: Identification of genetic interactions with priB links the PriA/PriB DNA replication restart pathway to double-strand DNA break repair in Escherichia coli
Source: G3 (Bethesda). 2022 Nov 3;12(12):jkac295. doi: 10.1093/g3journal/jkac295 (PMC9713433; doi:10.1093/g3journal/jkac295)
Supplement: jkac295_Supplemental_Material_Legends [file jkac295_supplemental_material_legends.docx]

**Figure S1.** ***Tn*-seq performed in Δ*priB*, *priC::kan*, and *priA300* *E. coli* strains.** (A) Volcano plot (generated with VolcaNoseR) for *Tn*-seq results in Δ*priB* cells. The fold-change (log_2_) in unique insertions within each gene (in *wt* vs Δ*priB* strains) is plotted against the probability of essentiality p-value adjusted for multiple comparisons (-log_10_) (Burger et al. 2017). Genes exceeding the fold-change (>4) and significance (>4) thresholds are colored blue and labeled. The *rdgB* gene is also labeled. Circos plots depicting the results of the *Tn*-seq screens in (B) *priC::kan* and (C) *priA300* cells. Each bar in the Circos plots represents the weighted read (log_10_) ratio of a single gene where extension into the blue or orange region corresponds to a detrimental or beneficial, respectively, effect of gene disruption. The *priA300* strain very poorly tolerated transposon insertions within *rep*. Genes with less than three average unique transposon-insertions per replicate in the *wt* condition were omitted.

**Figure S2.** **Effects of mutations on DNA damage sensitivity in *E. coli*.** The results contained in Figure 4 are expanded to reflect the viabilities of *priC*, *rdgC*, *rdgB*, *uup*, and *nagC* mutants plated on LB-agar with 0-30 ng/mL ciprofloxacin. A *recA* deletion strain was utilized as a positive control of ciprofloxacin hypersensitivity. Dilutions (from left to right) are 10x serial dilutions from normalized overnight culture. Displayed spot plate data are representative of three replicates.

**Figure S3.** **DSB formation in mutant *E. coli* strains.** (A) Representative images depicting MuGam-GFP (green) foci and FM 4-64-stained membranes (red) for SMR14334, *wt*, Δ*rep*, *lexA::kan*, *polA12(ts)*, Δ*dam*, Δ*priC*, Δ*rdgC*, Δ*uup*, Δ*nagC*, and Δ*rdgB* strains. Scale bars are 10 µm. The abundance of MuGam-GFP foci per cell (B), measured cell lengths (C), distribution of the number of foci per cell in cells with MuGam-GFP foci (D), and mean fluorescence per cell (E) are shown for all strains included in A. (B-D) Mean values are depicted with error bars representing standard error of the mean. (E) Median values are depicted as gray or black bars. (B-C) Statistical significance (U-Mann-Whitney) for each strain compared to the *wt* control is displayed: P < 0.05 (*), P < 0.01 (**), P < 0.001 (***), and P < 0.0001 (****).

**Table S1.** **Strains used in this study.**

**Table S2.** **Oligonucleotides and plasmids used in this study.** The “*” indicates phosphorothioate bonds. The underlined bases in oAMrev reflect twelve distinct indexes (and primers) employed for multiplexing during sequencing. Italicized bases in oAM192/oAM193 and oAM215/oAM216 directed FRTkanFRT chromosomal insertion location to construct AM354 and AM395 strains, respectively.

**File S1. Analysis of Δ*priB*, *priC::kan*, and *priA300* *Tn*-seq data.**

**File S2. Colony counts for *priB*-pRC7 retention assays and growth competitions.**

**File S3. Fluorescence and brightfield microscopy data/analysis.**
