# Supplementary figures and images for: Identification of genetic interactions with priB links the PriA/PriB DNA replication restart pathway to double-strand DNA break repair in Escherichia coli
Source: G3 (Bethesda). 2022 Nov 3;12(12):jkac295. doi: 10.1093/g3journal/jkac295 (PMC9713433; doi:10.1093/g3journal/jkac295)

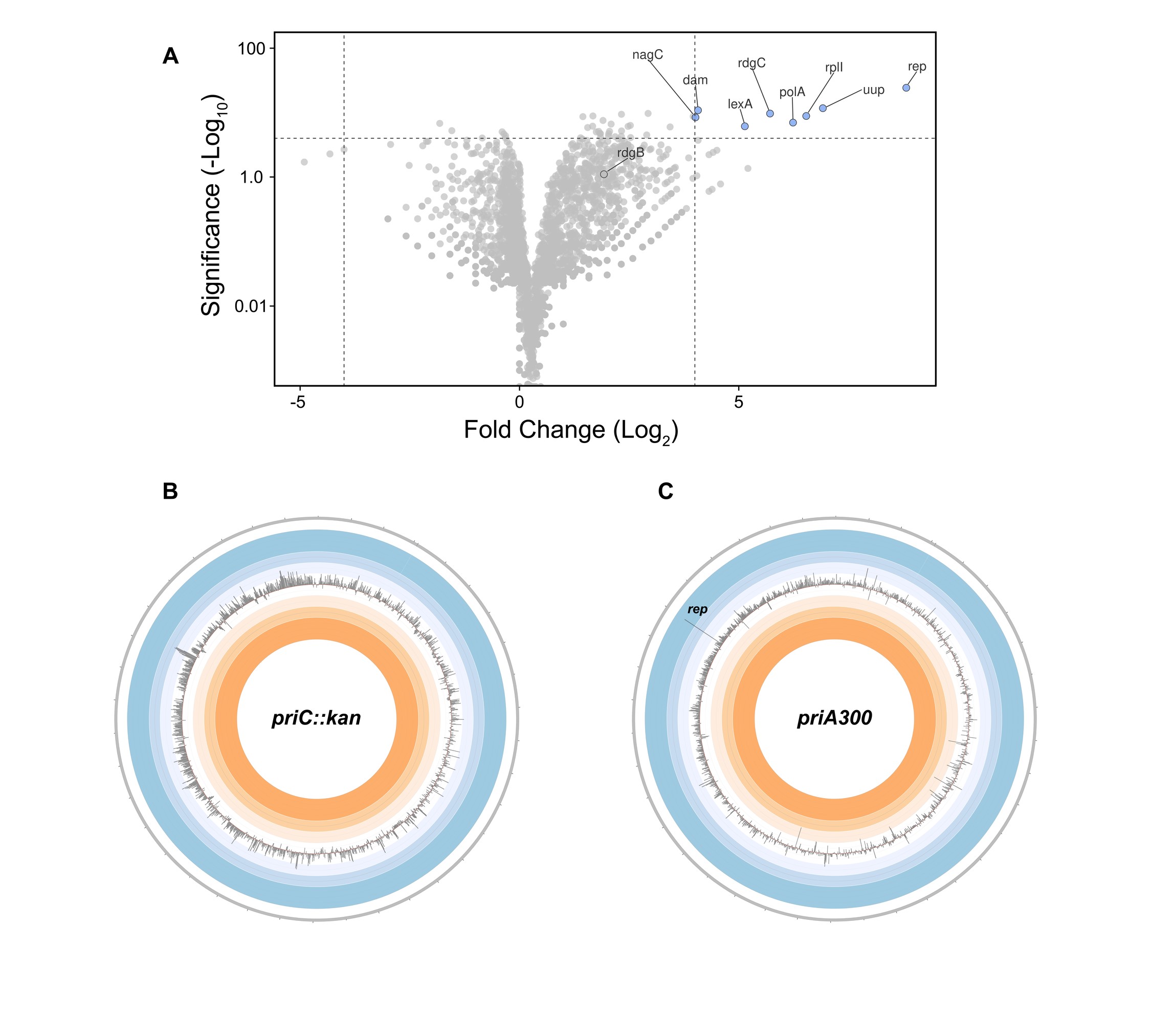

Supplement: jkac295_Supplementary_Figure_S1 [file jkac295_supplementary_figure_s1.jpeg]

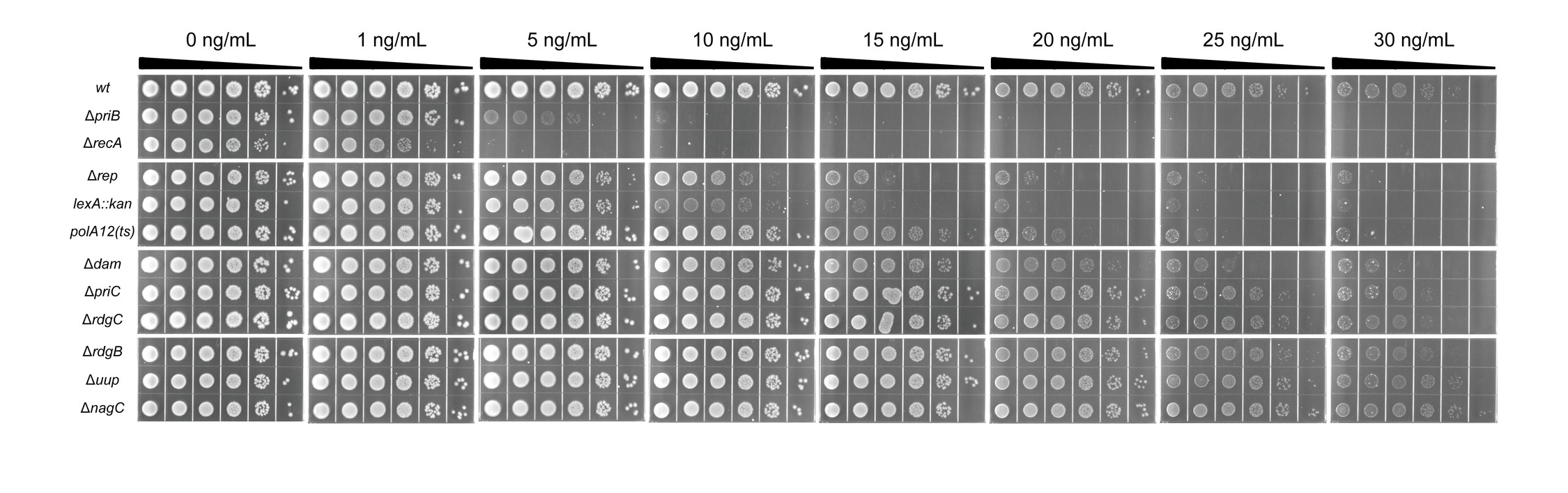

Supplement: jkac295_Supplementary_Figure_S2 [file jkac295_supplementary_figure_s2.jpeg]

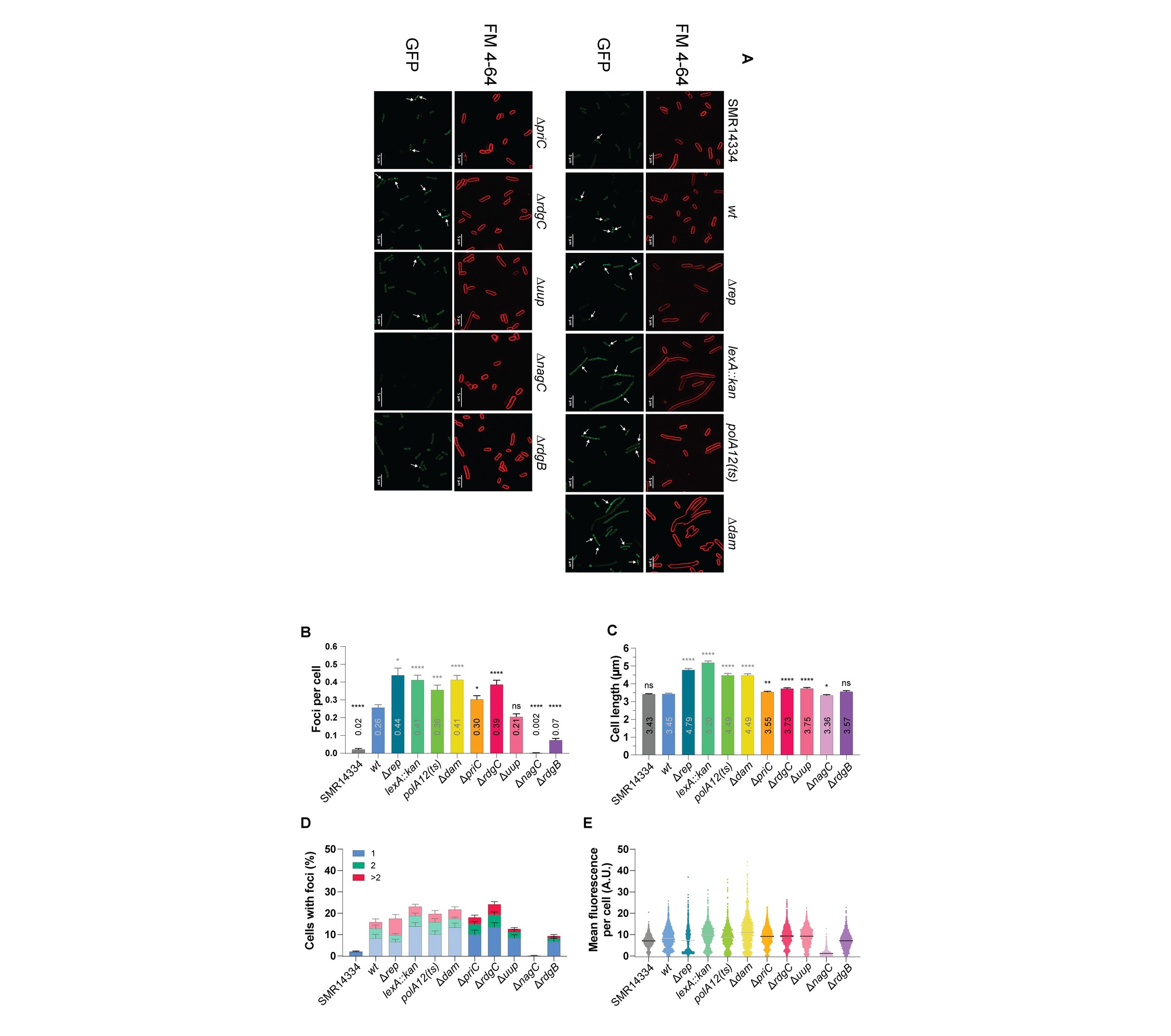

Supplement: jkac295_Supplementary_Figure_S3 [file jkac295_supplementary_figure_s3.jpeg]
